# Identification of 15 lncRNAs Signature for Predicting Survival Benefit of Advanced Melanoma Patients Treated with Anti-PD-1 Monotherapy

**DOI:** 10.3390/cells10050977

**Published:** 2021-04-22

**Authors:** Jian-Guo Zhou, Bo Liang, Jian-Guo Liu, Su-Han Jin, Si-Si He, Benjamin Frey, Ning Gu, Rainer Fietkau, Markus Hecht, Hu Ma, Udo S. Gaipl

**Affiliations:** 1 Department of Oncology, The Second Affiliated Hospital of Zunyi Medical University, Zunyi 563000, China; jianguo.zhou@fau.de (J.-G.Z.); sisihe1219@163.com (S.-S.H.); 2 Department of Radiation Oncology, Universitätsklinikum Erlangen, 91054 Erlangen, Germany; benjamin.frey@uk-erlangen.de (B.F.); rainer.fietkau@uk-erlangen.de (R.F.); markus.hecht@uk-erlangen.de (M.H.); 3 Comprehensive Cancer Center Erlangen-EMN, 91054 Erlangen, Germany; 4 Nanjing University of Chinese Medicine, Nanjing 210029, China; liangbo2018@126.com; 5 Special Key Laboratory of Oral Diseases Research, Stomatological Hospital Affiliated to Zunyi Medical University, Zunyi 563000, China; 13087891001@163.com (J.-G.L.); doctorjin1991@163.com (S.-H.J.); 6 Nanjing Hospital of Chinese Medicine Affiliated to Nanjing University of Chinese Medicine, Nanjing 210029, China; guning@njucm.edu.cn

**Keywords:** lncRNA, advanced melanoma, predictor, survival benefit, immune checkpoint inhibitor, PD-1, WGCNA, LASSO

## Abstract

The blockade of programmed cell death protein 1 (PD-1) as monotherapy has been widely used in melanoma, but to identify melanoma patients with survival benefit from anti-PD-1 monotherapy is still a big challenge. There is an urgent need for prognostic signatures improving the prediction of immunotherapy responses of these patients. We analyzed transcriptomic data of pre-treatment tumor biopsies and clinical profiles in advanced melanoma patients receiving only anti-PD-1 monotherapy (nivolumab or pembrolizumab) from the PRJNA356761 and PRJEB23709 data sets as the training and validation cohort, respectively. Weighted gene co-expression network analysis was used to identify the key module, then least absolute shrinkage and selection operator was conducted to determine prognostic-related long noncoding RNAs (lncRNAs). Subsequently, the differentially expressed genes between different clusters were identified, and their function and pathway annotation were performed. In this investigation, 92 melanoma patients with complete survival information (51 from training cohort and 41 from validation cohort) were included in our analyses. We initiallyidentified the key module (skyblue) by weighted gene co-expression network analysis, and then identified a 15 predictive lncRNAs (AC010904.2, LINC01126, AC012360.1, AC024933.1, AL442128.2, AC022211.4, AC022211.2, AC127496.5, NARF-AS1, AP000919.3, AP005329.2, AC023983.1, AC023983.2, AC139100.1, and AC012615.4) signature in melanoma patients treated with anti-PD-1 monotherapy by least absolute shrinkage and selection operator in the training cohort. These results were then validated in the validation cohort. Finally, enrichment analysis showed that the functions of differentially expressed genes between two consensus clusters were mainly related to the immune process and treatment. In summary, the 15 lncRNAs signature is a novel effective predictor for prognosis in advanced melanoma patients treated with anti-PD-1 monotherapy.

## 1. Introduction

Melanoma is one of the most aggressive malignant skin tumors, and the incidence has been increasing worldwide in recent decades [1,2]. Since the US Food and Drug Administration (FDA) and European Medicines Agency (EMA) approved a variety of immune checkpoint inhibitors (ICI) therapies for advanced melanoma in 2011, the overall mortality of advanced melanoma fell by 17.9% from 2013 to 2016 [3], but it is still at a very high level [4].

During the last years, biomarkers for effectiveness of tumor immunotherapy, including genomic instability as described by microsatellite instable (MSI) or tumor mutational burden (TMB) status, and immune cell infiltration into tumors, have been defined [5]. As one of the cancer types with high TMB and high immune cell infiltration, melanoma can be considered for immunotherapy [5]. Despite all efforts of early diagnosis, metastatic melanoma still has a poor prognosis and remains a challenge for treating physicians. Existing ICI therapies include the blockade of programmed cell death protein 1 (PD-1)/programmed death-ligand 1 (PD-L1) and the cytotoxic T-lymphocyte antigen 4 (CTLA-4) pathway. Therapies based on blockade of PD-1 in human melanoma achieved a success [6,7]; nivolumab (an anti-PD-1 monoclonal antibody) [8], and pembrolizumab (another anti-PD-1 monoclonal antibody) [9] monotherapy improve relapse/recurrence-free survival of stage III melanoma patients. Moreover, anti-PD-1 monoclonal antibodies indicate superior overall/recurrence-free survival versus anti-CTLA-4 agent for advanced melanoma [8,10,11]. Melanoma patients treated with anti-PD-1 monotherapy have a longer progression-free survival (PFS) compared to those treated with both strategies [12]. Further, anti-PD-1 monotherapy provides survival benefits in responding patients, but still many patients fail to respond [12]. Identifying the responsive population is a priority to optimize drug selection and improve patient outcomes. Future research should focus on identifying additional biomarkers to select patients who are most likely to benefit from certain immunotherapies.

Numerous long noncoding RNAs (lncRNAs) have been identified in human genomes [13] and they are nowadays considered as prognosis signatures in various tumors [14,15]. However, there has been limited systematic characterization of these elements in melanoma, especially in melanoma patients treated with anti-PD-1 monotherapy. Here, we identified a 15 lncRNAs signature in advanced melanoma by weighted gene co-expression network analysis (WGCNA) and logistic least absolute shrinkage and selection operator (LASSO) in the training cohort (PRJNA356761) and validated it in the validation cohort (PRJEB23709) to determine and predict the response of melanoma patients receiving anti-PD-1 monotherapy.

## 2. Materials and Methods

### 2.1. Data Source and Processing

**Figure 1:**
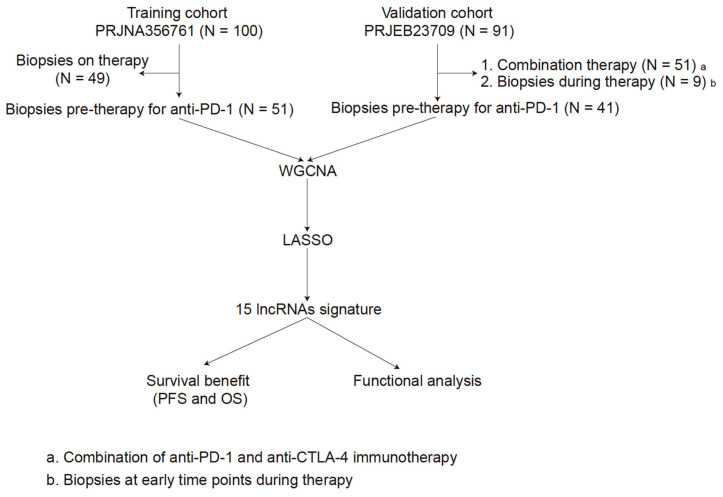
The overall workflow of this study.

The sequencing data of PRJNA356761 (training cohort) were obtained from https://github.com/riazn/bms038_analysis (accessed on 1 December 2020) [16]. The transcriptomic profiling of PRJEB23709 [12] (validation cohort) were obtained from the European Molecular Biology Laboratory-European Bioinformatics Institute (EMBL-EBI) database [17] (https://www.ebi.ac.uk/ena/data/view/PRJEB23709 (accessed on 1 December 2020)). Raw RNA-seq reads were aligned to the reference genome (UCSC hg38 with annotations from GRCh38.p13) using STAR [18] (v.2.5.3a). Gene expression was subsequently quantified using RSEM [19] (v.1.3.1). The lncRNA annotations were performed by gencode.v34.annotation (https://www.gencodegenes.org/human/ (accessed on 1 Feburary 2021)). The inclusion criteria were as follows: (1) advanced melanoma; (2) only anti-PD-1 monotherapy (nivolumab or pembrolizumab); (3) pre-treatment tumor biopsies. Moreover, only the inlier samples that were identified by hierarchical cluster analysis via the *hclust* function in WGCNA were included. The detailed process is shown in Figure 1.

### 2.2. Module Construction

After crossing all lncRNAs in the two cohorts, unsigned co-expression modules were constructed in the training cohort using the *WGCNA* algorithms in R as described previously [20]. We used one-step network construction method to identify co-expression modules through the *blockwiseModules* function in the *WGCNA* package [21]. Before co-expression network construction, the *flashClust* tool in R was utilized to perform hierarchical clustering analysis of the samples with the appropriate threshold value to detect and eliminate the outliers. According to scale-free topology criterion, a soft-thresholding power β (the power values ranged from 1 to 20), which was calculated by the *pickSoftThreshold* function of the *WGCNA*, was chosen to build an adjacency matrix [21]. In our study, the power of 6 was used for this network.

Then, the topological overlap matrix was constructed based on the adjacency matrix. A dissimilarity matrix was used to detect gene modules (gene sets with high topological overlap) through a dynamic tree cutting algorithm [22,23]. To obtain moderately sized modules, the minimum number of genes was set at 30 and a cutline was chosen to merge modules with similar expression patterns. To identify the relationships between modules and clinical traits, we calculated the correlation between module eigengenes and clinical trait and searched for the most significant associations. The module eigengenes was calculated by the first principal component, which were considered as a representative of the expression patterns of module genes [24]. For each module, we defined the module membership as the correlation of gene expression profile with module eigengene and the gene significance as the absolute value of the correlation between gene and clinical traits. In this study, genes with high module membership in a module were assigned to the module and the module with high gene significance and *p* value < 0.05 was considered to be highly related to clinical traits. Moreover, after introducing validation cohort, we used network-based statistics, which generated a composite statistic value (Z_summary_) using a permutation test to measure the strength of lncRNAs module and expression module preservation, to assess whether the density and connectivity patterns of lncRNAs were also preserved [25]. Z_summary_ > 5 implies strong evidence for module preservation [26,27]. Since the Z_summary_ statistic bias towards a module with a large size [25], a rank-based statistic medianRank, calculated from observed preservation values and conducted no permutation test against background gene modules, was used to measure the relative preservation irrespective of module size [28].

### 2.3. Identifcation of lncRNAs Signature

Although which module was most relevant to the predictor of melanoma can be identified after WGCNA, we further applied a novel, modern statistical shrinkage technique to examine the association between lncRNAs and the prognosis of melanoma to establish prognostic lncRNAs signature. The logistic LASSO regression model is a shrinkage method that can actively select from a large and potentially multicollinear set of variables in the regression, resulting in a more relevant and interpretable set of predictors [29]. One interesting property of LASSO is that the estimates of the regression coefficients are sparse, which means that many components are exactly 0 [30]. We utilized the *glmnet* package (version 2.0–16) to fit the logistic LASSO regression.

### 2.4. Development and Validation of the lncRNAs Signature

After the identification of the predictive module, we further clustered melanoma population into different consensus clusters though an optimum cutoff value identified by *survivalROC* package (version 1.0.3). Ultimately, the lncRNAs signature can distinguish different consensus clusters, and the *survival* package was applied to perform Kaplan–Meier analysis with the log-rank test to analyze the overall survival (OS) and PFS in both training and validation cohorts. Moreover, we used a time-dependent receiver operating characteristic (ROC) curve to assess the survival prediction, and the area under the ROC curve (AUC) value was computed with the *ROCR* package (version 1.0.–7) to measure prognostic or predictive accuracy, as described previously [31]. In addition, we calculated the response rates of anti-PD-1 therapy based on the lncRNAs signature in both, training and validation cohort.

### 2.5. Functional Analysis

Subsequently, the differentially expressed genes (DEGs) between different consensus clusters distinguished by the lncRNAs signature were identified by *limma* package [32] (version 3.28.14) with the cutoff of *p* < 0. Functional analysis were performed using the *clusterProfiler* package [33] (version 3.18.0) and *msigdbr* package [34] (version 7.2.1) to expand our understanding of those lncRNAs signature-related functions and their coordinated regulatory networks.

### 2.6. Immune Cell Enrichment Analysis by xCell

Immune cell enrichment analysis was conducted by *xCell* function [35] in *immunedeconv* package (version 2.0.3) [36].

## 3. Results

### 3.1. Patient Characteristics

In total, 100 tumor biopsies from melanoma patients treated with anti-PD-1 monotherapy (nivolumab) were assessed by whole-exome sequencing in PRJNA356761, among them, 49 biopsies were pre-therapy [16]. PRJEB23709 included 158 tumor biopsies from 120 melanoma patients treated with anti-PD-1 monotherapy (nivolumab or pembrolizumab) or combined anti-PD-1 and anti-CTLA-4 (nivolumab or pembrolizumab combined with ipilimumab) [12]. In our study, we included 51 melanoma patients form PRJNA356761 and 41 melanoma patients form PRJEB23709 (Figure 1). The patient characteristics are listed in Table 1. Responders were defined as patients with a Response Evaluation Criteria in Solid Tumors (RECIST) response [37] of complete response (CR), partial response (PR), or stable disease (SD) of greater than 6 months with no progression, and non-responders as progressive disease (PD) or SD for less than or equal to 6 months before disease progression.

### 3.2. WGCNA and Key Module Identification

**Figure 2:**
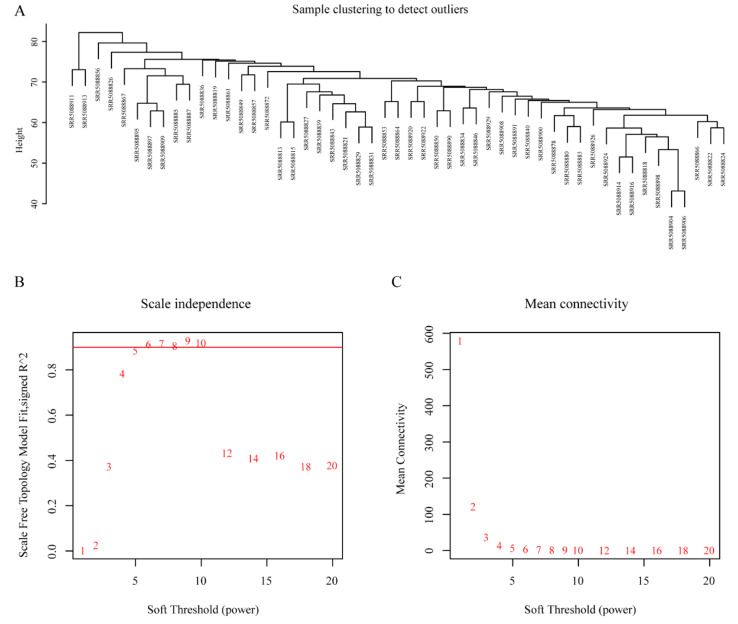
Sample clustering dendrogram and determination of soft-thresholding power in weighted gene co-expression network analysis (WGCNA). (**A**) Sample clustering dendrogram to detect outliers. (**B**) Analysis of the scale-free fit index for various soft-thresholding power. (**C**) Analysis of the mean connectivity for various soft-thresholding powers.

**Figure 3:**
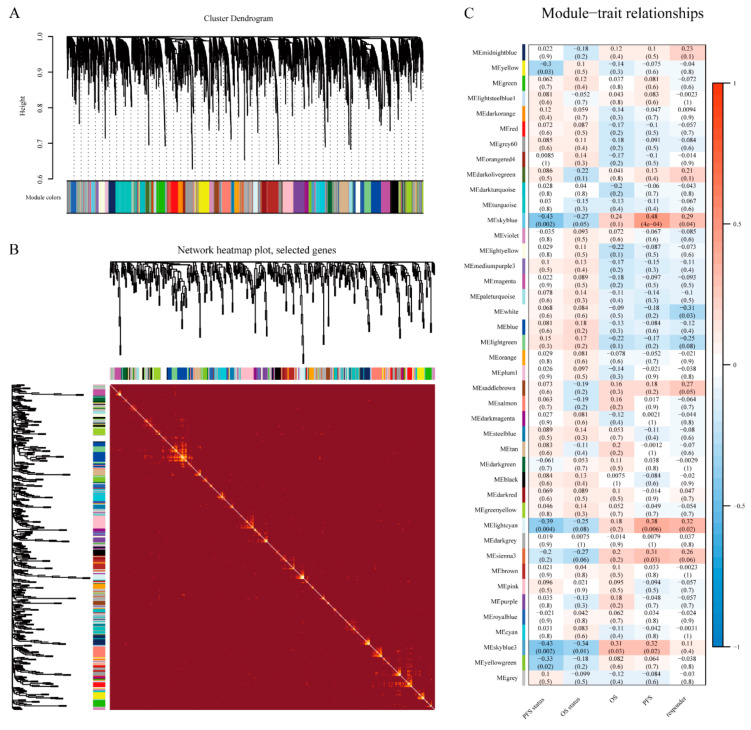
Identification of key module related to survival benefit (progression-free survival (PFS) and overall survival (OS)) by WGCNA. (**A**) Clustering dendrogram of long noncoding RNAs (lncRNAs) with dissimilarity based on topological overlap together and assigned module colors. (**B**) The heatmap plot of visualizing all modules. (**C**) The module-trait heatmap plot.

We both obtained data of 16,899 lncRNAs from the training and validation cohorts, by transcriptome analysis, and 4653 lncRNAs after the intersection were included to construct co-expression networks by WGCNA. To exclude the outliers, we chose 85 for the cut tree height for the samples (Figure 2A), and 48 samples were included for subsequent analysis. Then we identified the soft-thresholding power β of six to construct a scale-free network (Figure 2B,C). As a result, 12 co-expression modules were identified by gathering similarly expressed lncRNAs (Figure 3A). Interactions between 12 modules were subsequently analyzed. The heatmap demonstrated the topological overlap matrix among all of 4653 lncRNAs in our study (Figure 3B), indicating that each module showed independent validation to each other. Then, the correlations between module eigengene, and clinical traits were discovered. Moreover, after introducing the validation cohort, we plotted the preservation median rank and Z_summary_ for the modules as a function of module size. The 12 modules (gold, yellow, pink, turquoise, magenta, green, lightgreen, white, darkred, skyblue, skyblue3, and lightsteelblue1) showed strong evidence of preservation (Z_summary_ > 5) (Figure S1). The module eigengenes in the skyblue module showed a higher correlation with PFS status, OS status, OS, PFS, and responder (R_PFS status_^2^ = −0.43, *p* < 0.002; R_OS status_^2^ = −0.27, *p* < 0.05; R_OS_^2^ = 0.24, *p* < 0.1; R_PFS_^2^ = 0.48, *p* < 0.0001; R_responder_^2^ = 0.29, *p* = 0.04; respectively) (Figure 3C). We therefore chose the skyblue module for further analyses.

### 3.3. Identification of lncRNAs Signature

There were 63 lncRNAs in the skyblue prognosis module we identified (Table S1). Though the logistic LASSO regression model, we shrank to 15 lncRNAs (AC010904.2, LINC01126, AC012360.1, AC024933.1, AL442128.2, AC022211.4, AC022211.2, AC127496.5, NARF-AS1, AP000919.3, AP005329.2, AC023983.1, AC023983.2, AC139100.1, and AC012615.4) to regard as lncRNAs signature (Table 2).

### 3.4. Development and Validation of the lncRNAs Signature

**Figure 4:**
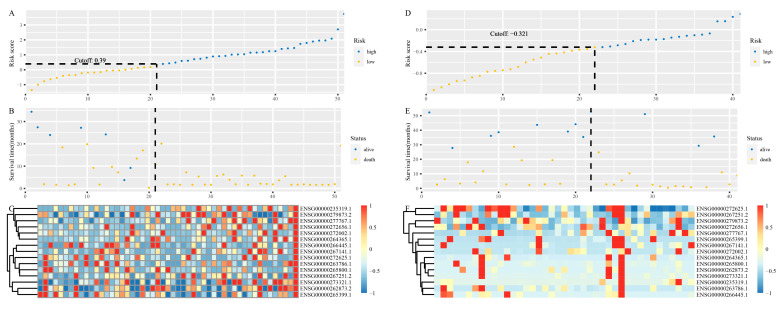
Characteristics of the 15 lncRNAs signature. (**A**) The risk score of each melanoma patient in the training cohort. (**B**) PFS and survival status of patients in the training cohort. (**C**) Heat map of gene expression profiles of melanoma patients in the training cohort. (**D**) The risk score of each melanoma patient in the validation cohort. (**E**) PFS and survival status of patients in the validation cohort. (**F**) Heat map of gene expression profiles of melanoma patients in the validation cohort.

**Figure 5:**
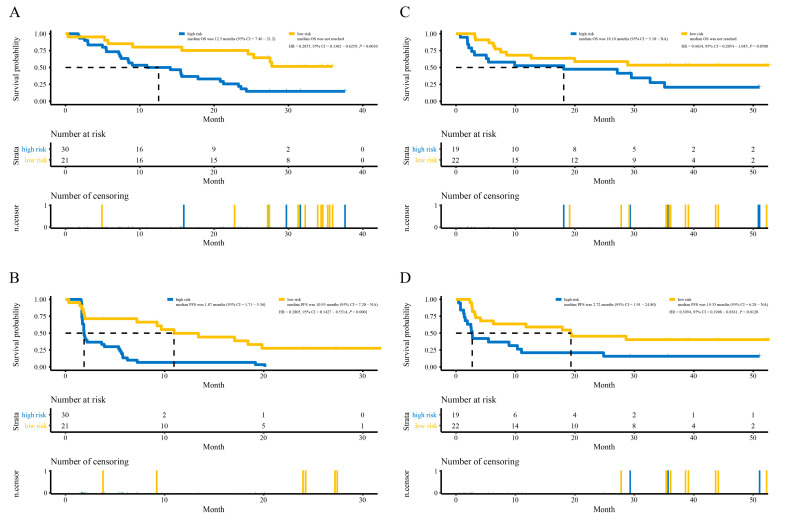
The survival curves of OS and PFS. (**A**) The survival curve of OS in the training cohort. (**B**) The survival curve of PFS in the training cohort. (**C**) The survival curve of OS in the validation cohort. (**D**) The survival curve of PFS in the validation cohort.

On the basis of the time-dependent ROC curve analysis, the optimal cutoff value that could be used for the 15 lncRNAs signature to stratify melanoma patients treated with anti-PD-1 monotherapy into the high- or low-risk group was determined to be 0.39 in the training cohort (Figure 4A–C). All 51 patients in the training cohort were segregated into the high-risk group (*n* = 30) and the low-risk group (*n* = 21), and the low-risk group exhibited significantly better OS than the high-risk group (hazard ratio (HR) = 0.2855, 95% confidence interval (CI) = 0.1302~0.6259, *p* = 0.0010, Figure 5A). For the low-risk group, the median OS was not reached, whereas the median OS was 12.5 months (95% CI = 7.46~21.2) for the high-risk group (Figure 5A). Regarding PFS, all 51 patients in the training cohort were similarly segregated into the low- and high-risk groups, and the low-risk group was correlated with significantly favorable PFS (HR = 0.2805, 95% CI = 0.1427~0.5514, *p* = 0.0001, Figure 5B). For the low-risk group, the median PFS was 10.95 months (95% CI = 7.20~NA), whereas the median PFS was 1.87 months (95% CI = 1.71~5.36) for the high-risk group (Figure 5B).

To examine the robust and realistic application of the lncRNAs signature, the performance of the 15 lncRNAs signature was validated in the validation cohort (Figure 4D–F). The developed 15 lncRNAs signature could actively predict OS and PFS in melanoma patients treated with anti-PD-1 monotherapy in the validation cohort. The lncRNAs signature significantly stratified patients into low- and high-risk groups in terms of OS; more specifically, all 41 patients were segregated into the low-risk group (n = 22) and the high-risk group (n = 19) and showed significantly different OS rates (HR = 0.4634, 95% CI = 0.2054~1.045, *p* = 0.0580, Figure 5C) according to the optimum cutoff point (−0.321) acquired from the training cohort (Figure 4D). For the low-risk group, the median OS was not reached, whereas the median OS was 18.10 months (95% CI = 5.10~NA) for the high-risk group (Figure 5C). Concerning PFS, the low-risk group tended to favor favorable PFS (HR = 0.3994, 95% CI = 0.1908~0.8361, *p* = 0.0120, Figure 5D). For the low-risk group, the median PFS was 19.33 months (95% CI = 6.28~NA), whereas the median PFS was 2.72 months (95% CI = 1.91~24.80) for the high-risk group (Figure 5D). Overall, the 15 lncRNAs signature appears to independently estimate OS and PFS in melanoma patients treated with anti-PD-1 monotherapy well.

Time-dependent ROC curve analysis was performed to compare the sensitivity and specificity of the prediction of OS and PFS with the 15 lncRNAs signature in the training and validation cohorts. AUC values at 12, 18, and 24 months obtained from ROC curve analysis were used to assess the prognostic accuracy.

**Figure 6:**
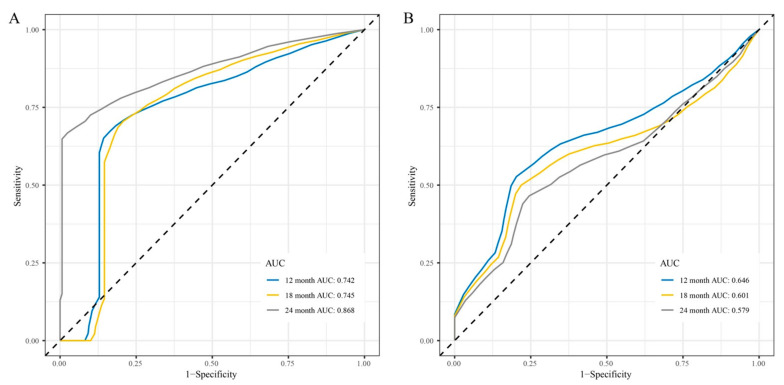
Time-dependent receiver operating characteristic (ROC) curves for PFS predicted with the 15 lncRNAs signature. (**A**) Time-dependent ROC curves for PFS predicted with the 15 lncRNAs signature in the training cohort. (**B**) Time-dependent ROC curves for PFS predicted with the 15 lncRNAs signature in the validation cohort.

In the training cohort, the 15 lncRNAs signature reached 12-month AUC values of 0.636, 18-month AUC values of 0.651, and 24-month AUC values of 0.746 for OS (Figure S2A). The validation cohort was characterized by 12-month AUC values of 0.527, 18-month AUC values of 0.485, and 24-month AUC values of 0.490 for OS (Figure S2B). However, regarding PFS, both, the training and the validation cohort had good AUC values, namely 12-month AUC values of 0.742, and 0.646, 18-month AUC values of 0.745 and 0.601, and 24-month AUC values of 0.868 and 0.579, respectively (Figure 6).

### 3.5. Response Rates Based on the lncRNAs Signature

**Figure 7:**
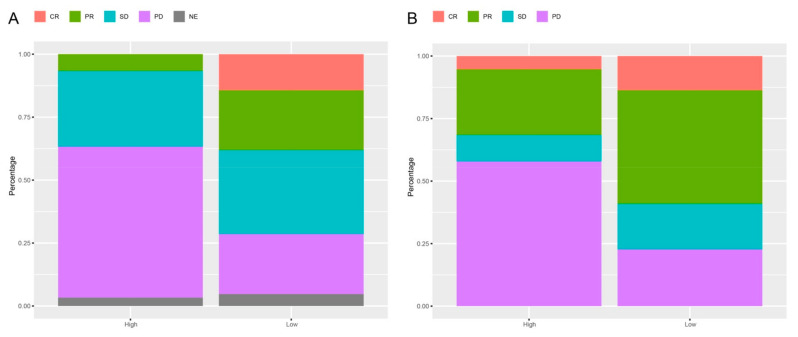
Response rates in the high-risk (high) versus the low-risk (low) groups in advanced melanoma patients treated with anti- PD-1 monotherapy based on the 15 lncRNAs signature. (**A**) Response rates based on the 15 lncRNAs signature in advanced melanoma patients treated with anti-PD-1 monotherapy in the training cohort. (**B**) Response rates based on the 15 lncRNAs signature in advanced melanoma patients treated with anti-PD-1 monotherapy in the validation cohort. CR: complete response; PR: partial response; SD: stable disease; PD: progressive disease.

Among advanced melanoma patients treated with anti-PD-1 monotherapy, there was a strong association between best overall response and the identified 15 lncRNAs signature. In the training cohort, 2 (7%) of 30 patients in the high-risk group versus 8 (38%) of 21 patients in the low-risk group had an objective response (CR or PR); CR were achieved by 0 (0%) of 30 patients in the high-risk group versus 3 (14%) of 21 patients in the low-risk group; 11 (37%) of 30 patients in the high-risk group had disease control (CR, PR, or SD) as their best overall response, compared to 15 (71%) of 21 patients in the low-risk group (*p* = 0.0236, Figure 7).

In the validation cohort, 6 (32%) of 19 patients in the high-risk group versus 13 (59%) of 22 patients in the low-risk group had an objective response; CR were achieved by 1 (5%) of 19 patients in the high-risk group versus 3 (14%) of 22 patients in the low-risk group; 8 (42%) of 19 patients in the high-risk group had best overall response compared to 17 (77%) of 22 patients in the low-risk group (*p* = 0.0476, Figure 7).

### 3.6. Functional Analysis

**Figure 8:**
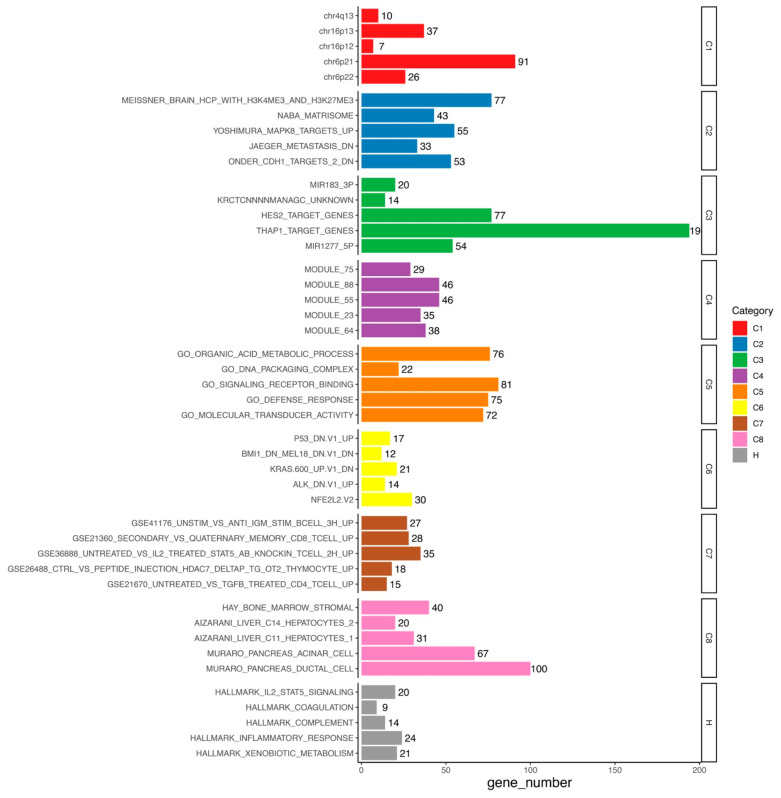
Functional enrichment of DEGs.

We identified 4709 differentially expressed genes (DEGs) in the high-risk versus low-risk group, including 2593 up-regulated and 2116 down-regulated DEGs (Table S2). The Molecular Signatures Database (MSigDB) gene sets are divided into nine major collections: H (hallmark gene sets), C1 (positional gene sets), C2 (curated gene sets), C3 (regulatory target gene sets), C4 (computational gene sets), C5 (ontology gene sets), C6 (oncogenic signature gene sets), C7 (immunologic signature gene sets), and C8 (cell type signature gene sets) [34,38]. According to MSigDB database (version 7.2) which was updated in September 2020, further enrichment analysis showed that these 4709 DEGs were enriched to IL2-STAT5 signaling, inflammatory response, and xenobiotic metabolism in H, MAPK8 targets, jaeger metastasis dn, and onder cdh1 targets 2 dn in C2, miR183-3p, HES2, THAP1, and miR1277-5p in C3, organic acid metabolic process, DNA packaging complex, signaling receptor binding, defense response, and molecular transducer activity in C5, P53, BMI1, KRAS, ALK, and NFE2L2 in C6, B cell, CD8^+^ T cell, and CD4^+^ T cell in C7, and hay bone marrow stromal, aizarani liver C14 hepatocytes 2, aizarani liver C11 hepatocytes 1, muraro pancreas acinar cell, and muraro pancreas ductal cell in C8 (Figure 8, Table S3).

### 3.7. Immune Cell Enrichment Analysis

A compendium of 39 cell types, comprising multiple adaptive and innate immune cells derived from thousands of expression profiles, was identified in the training cohort with xCell, a novel method that integrates the advantages of gene set enrichment with deconvolution approaches [35]. We identified CD4+ Th1 cells and CD8+ naïve T cells to be significantly altered (*p* = 0.0103 and 0.0436, respectively; Table S4).

## 4. Discussion

Over the past decades, with a deeper understanding of the pathophysiology and the manifold roles of the immune system in cancer management [39], ICI have made their way into clinics as single treatment or in multimodal settings for several tumor entities [40,41,42]. While these advances in the treatment of metastatic melanoma have improved responses and survival [43], still the majority of patients do not respond properly or respond with side effects to ICI. Although the regimens of ICI-based immunotherapy have been continuously adjusted and optimized [44,45,46], patients with melanoma still have heterogeneous ICI response, especially to anti-PD-1 monotherapy. This requires screening for the most sensitive subgroups. An early assessment, especially at the pre-treatment stage, for anti-PD-1 monotherapy responses by predictive signature is crucial for the selection of patients who are most likely to benefit from anti-PD-1 monotherapy. Hence, in this study, we combined WGCNA and LASSO to discover 15 lncRNAs predictor for patients with advanced melanoma, which can also reflect the anti-PD-1 monotherapy response, and then initially explored the potential mechanisms.

Hence, in this study, we succeeded to identify a 15 lncRNAs predictor to response to anti-PD-1 monotherapy for patients with melanoma. A total of 15 lncRNAs (AC010904.2, LINC01126, AC012360.1, AC024933.1, AL442128.2, AC022211.4, AC022211.2, AC127496.5, NARF-AS1, AP000919.3, AP005329.2, AC023983.1, AC023983.2, AC139100.1, and AC012615.4) signature was identified by computational approach with datasets of a training and a validation cohort. All data of patients included in our analyses were based on anti-PD-1 monotherapy.

The survival curves indicate that the identified 15 lncRNAs signature distinguishes patients who are most likely to have survival benefit of anti-PD-1 monotherapy, regardless of PFS and OS. However, the signature was stronger for prediction of PFS when being validated. This is of high value, since OS might be generally influenced by non-disease specific events.

Furthermore, we used the DEGs between two consensus clusters as a starting point to get first hints why there is a survival benefit between the two clusters distinguished by the 15 lncRNAs signature by using MSigDB, which is has been seldomly performed in previous studies [47,48]. We found several immune-related cells, processes, and pathways being affected. Recent studies showed that IL2-STAT5 signaling pathway is closely related to immunity [49,50]. The activation of the MAPK8 in melanoma could trigger the massive functional natural killer cells infiltration [51]. Moreover, the enriched C2 contents jaeger metastasis dn is related to the molecular mechanisms of malignant melanoma progression and metastasis [52], and onder cdh1 targets 2 dn contributes to metastatic dissemination [53]. In C7, we also identified several immune-related cells, such as B cell, CD8^+^ T cell, and CD4^+^ T cell. Additionally, the identified pathways were also enriched for PD-L1/PD-1 axis events, which is exactly the target of anti-PD-1 therapy. In terms of adaptive immune response, CD4^+^ T cells are regarded as important factors regulating immune balance [54]. In the xcell analysis, we also highlighted CD4^+^ T cell and CD8^+^ T cell, which is consistent with functional analysis. T cells are prominent TILs in melanoma [55]. CD4^+^ T cells are associated with anti-tumor responses in melanoma [56,57,58]; CD8^+^ T cells also play a role herein [59]. Further, different CD8^+^ T cell subpopulations have predictive value in melanoma [60].

Moreover, similar to previous studies [61,62,63], we also succeeded with the identified 15 lncRNAs signature to stratify patients in the high- and low-risk groups. The high-risk group had a lower best overall response compared to the low-risk group, both in the training and in the validation cohort (Figure 7), indicating the robustness of the 15 lncRNAs signature. Therefore, one can hypothesize that the identified 15 lncRNAs signature might affect the survival benefit of advanced melanoma patients treated with anti-PD-1 monotherapy through generally immune-related cells, processes, and pathways.

The lncRNAs we identified may provide new ideas and insights for predicting the survival benefit of melanoma patients who receive anti-PD-1 monotherapy. However, one has to consider that our analyses are based on the publicly available dataset. Thus, we could not obtain all the clinic-pathological characteristics for each patient. Furthermore, we only included data of pre-treatment tumor biopsies from melanoma patients treated with anti-PD-1 monotherapy.. Although we used two completely independent data as the training cohort and the validation cohort, anti-PD-1 monotherapy in the two data is not exactly consistent. Moreover, since the population we included in this retrospective study was melanoma patients receiving anti-PD-1 monotherapy with complete follow-up data, our sample size was relatively small. Therefore, further testing and verifying of the identified 15 lncRNAs signature in prospective studies will be necessary.

## 5. Conclusions

However, we succeeded to characterize lncRNA expression profiles to identify particularly PFS benefit of melanoma patients receiving anti-PD-1 monotherapyOur analyses provide also hints that this signature affects the response of melanoma patients treated with anti-PD-1 monotherapy by influencing immune-related pathways. The 15 lncRNAs signature is therefore a novel predictor for survival in melanoma patients treated with anti-PD-1 monotherapy.

## Figures and Tables

**Table 1:** Characteristics of patients in the training and validation cohorts.

Features	Training Cohort PRJNA356761 (N = 51)	Validation Cohort PRJEB23709 (N = 41)
Gender	NA	
Male		26 (63%)
Female		15 (37%)
Age	NA	
≥60		26 (63%)
<60		15 (37%)
RECIST Response		
CR	3 (6%)	4 (10%)
PR	7 (14%)	15 (36%)
SD	16 (31%)	6 (15%)
PD	23 (45%)	16 (39%)
NE	2 (4%)	0 (0%)
Survival time		
PFS (days)	111 (52~288)	271 (80~891)
OS (days)	484 (220~836)	607 (169~1085)
Progressed		
Yes	26 (51%)	29 (71%)
No	25 (49%)	12 (29%)
Status		
Alive	17 (33%)	17 (41%)
Dead	34 (67%)	24 (59%)

Note: Data were shown as N (%) or median (Q1~Q3). RECIST: Response Evaluation Criteria in Solid Tumors, CR: complete response, PR: partial response, SD: stable disease, PD: progressive disease, NE: Not Evaluated, PFS: progression-free survival, OS: overall survival, and NA: Not applicable.

**Table 2:** Details of 15 lncRNAs signature.

ID	Name	Coefficient
ENSG00000272002.1	AC010904.2	−5.68483
ENSG00000279873.2	LINC01126	−2.91046
ENSG00000235319.1	AC012360.1	−3.83868
ENSG00000272656.1	AC024933.1	−5.09725
ENSG00000277767.1	AL442128.2	−3.06533
ENSG00000265800.1	AC022211.4	−5.29139
ENSG00000263786.1	AC022211.2	−5.69648
ENSG00000262873.2	AC127496.5	−3.43136
ENSG00000266445.1	NARF-AS1	−5.70092
ENSG00000272625.1	AP000919.3	−5.27859
ENSG00000265399.1	AP005329.2	−5.76167
ENSG00000264365.1	AC023983.1	−3.39892
ENSG00000273321.1	AC023983.2	−4.87733
ENSG00000267251.2	AC139100.1	−5.64345
ENSG00000267141.1	AC012615.4	−3.59529

## Data Availability

The raw data presented in this study are openly available in BioPrject (No.PRJNA356761) and EMBL-EBI (No. PRJEB23709).

## Supplementary Materials

The following are available online at https://www.mdpi.com/article/10.3390/cells10050977/s1, Figure S1: The median rank and Z_summary_ statistics of each module preservation, Figure S2: Time-dependent ROC curves for OS predicted with the 15 lncRNAs signature. (**A**) Time-dependent ROC curves for OS predicted with the 15 lncRNAs signature in the training cohort. (**B**) Time-dependent ROC curves for OS predicted with the 15 lncRNAs signature in the validation cohort. Table S1: Details of lncRNAs in the skyblue module, Table S2: Details of the DEGs, Table S3: Details of the DEGs function annotation analysis, Table S4: Immune cell enrichment analysis by xCell.
