# Supplementary material for: Identification of 15 lncRNAs Signature for Predicting Survival Benefit of Advanced Melanoma Patients Treated with Anti-PD-1 Monotherapy
Source: Cells. 2021 Apr 22;10(5):977. doi: 10.3390/cells10050977 (PMC8143567; doi:10.3390/cells10050977)
Supplement: Supplementary file 1 [file cells-10-00977-s001.zip › Zhou et al_R_Suppl Table4.docx]

| Immune and stromal cells | P-value |
| --- | --- |
| Th1 cells | 0.010313 |
| CD8+ naive T cell | 0.043602 |
| Hematopoietic stem cell | 0.123576 |
| Myeloid dendritic cell | 0.133298 |
| T cell NK | 0.157494 |
| Stroma score | 0.189365 |
| Monocyte | 0.216474 |
| CD4+ central memory T cell | 0.229213 |
| CD4+ naive T cell | 0.260862 |
| T cell regulatory Tregs | 0.274307 |
| Macrophage M1 | 0.326097 |
| NK cell | 0.329257 |
| Endothelial cell | 0.359717 |
| CD8+ effector memory T cell | 0.370786 |
| Common myeloid progenitor | 0.378099 |
| Macrophage | 0.389489 |
| Macrophage M2 | 0.406173 |
| CD4+ non regulatory T cell | 0.426011 |
| Eosinophil | 0.431722 |
| Th2 cells | 0.443792 |
| Granulocyte monocyte progenitor | 0.460197 |
| Microenvironment score | 0.467124 |
| Cancer associated fibroblast | 0.513803 |
| B cell naive | 0.516964 |
| B cell memory | 0.537071 |
| CD8+ T cell | 0.538466 |
| CD4+ effector memory T cell | 0.586828 |
| B cell | 0.603147 |
| Neutrophil | 0.667205 |
| CD8+ central memory T cell | 0.675462 |
| Mast cell | 0.70036 |
| Myeloid dendritic cell activated | 0.781132 |
| B cell plasma | 0.786457 |
| CD4+ memory T cell | 0.845127 |
| Common lymphoid progenitor | 0.858405 |
| Immune score | 0.863708 |
| T cell gamma delta | 0.870094 |
| Plasmacytoid dendritic cell | 0.93697 |
| Class switched memory B cell | 0.942993 |

Table S4: Immune cell enrichment analysis by xCell
