# Supplementary figures and images for: Identification of 15 lncRNAs Signature for Predicting Survival Benefit of Advanced Melanoma Patients Treated with Anti-PD-1 Monotherapy
Source: Cells. 2021 Apr 22;10(5):977. doi: 10.3390/cells10050977 (PMC8143567; doi:10.3390/cells10050977)

# Preservation Median rank

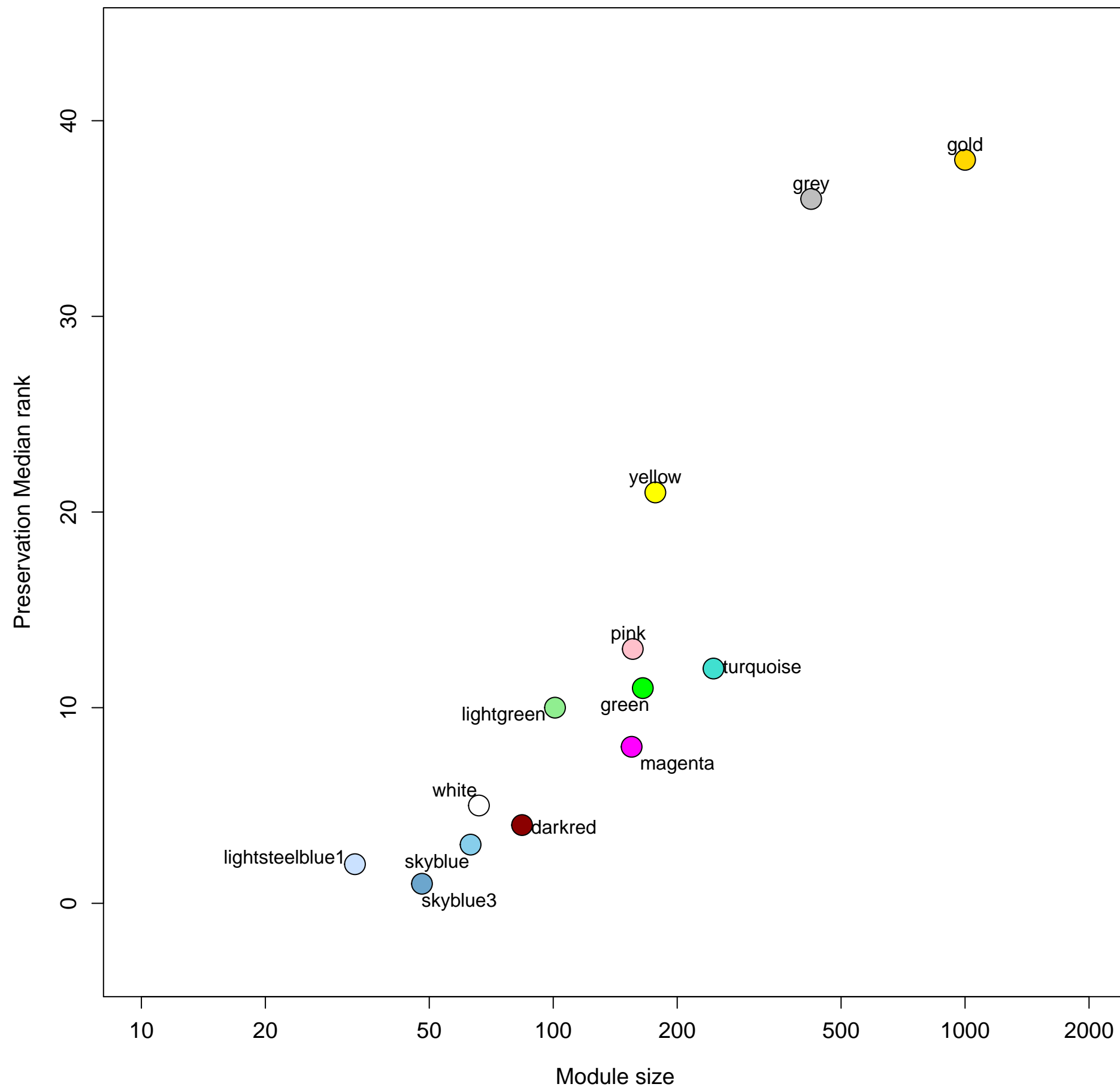

# Preservation Zsummary

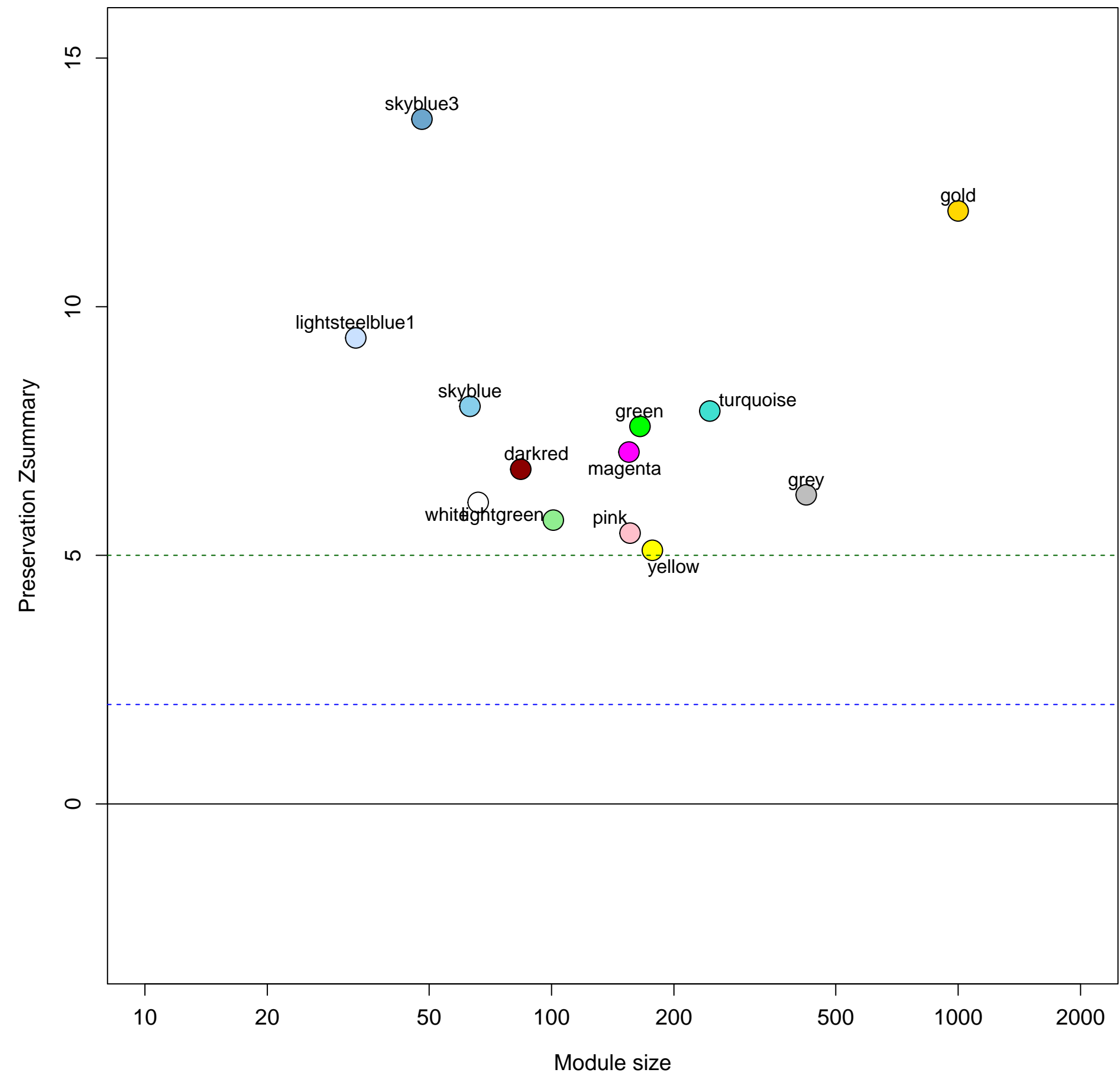

Supplement: Supplementary file 1 [file cells-10-00977-s001.zip › Zhou et al_R_Suppl Figure1.pdf]

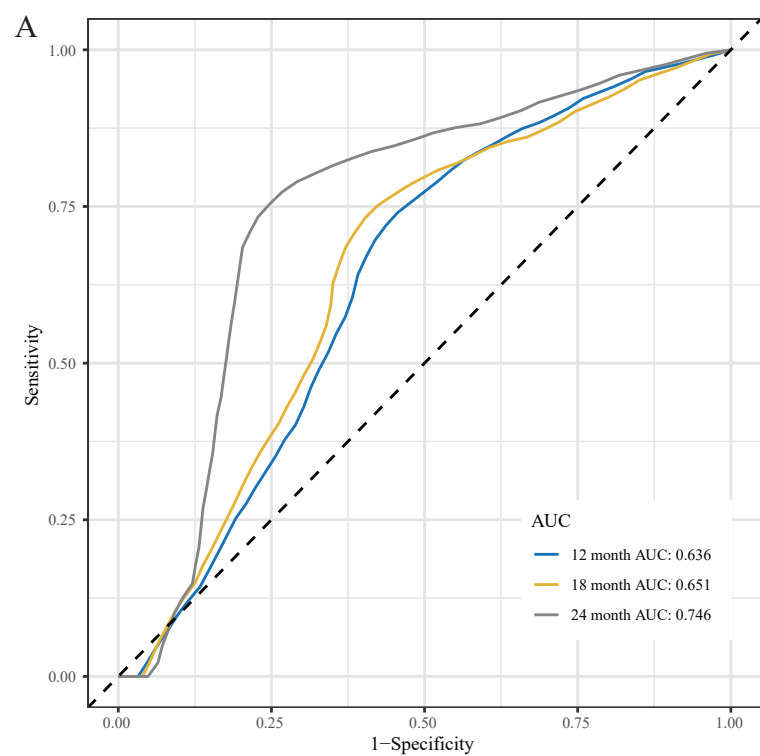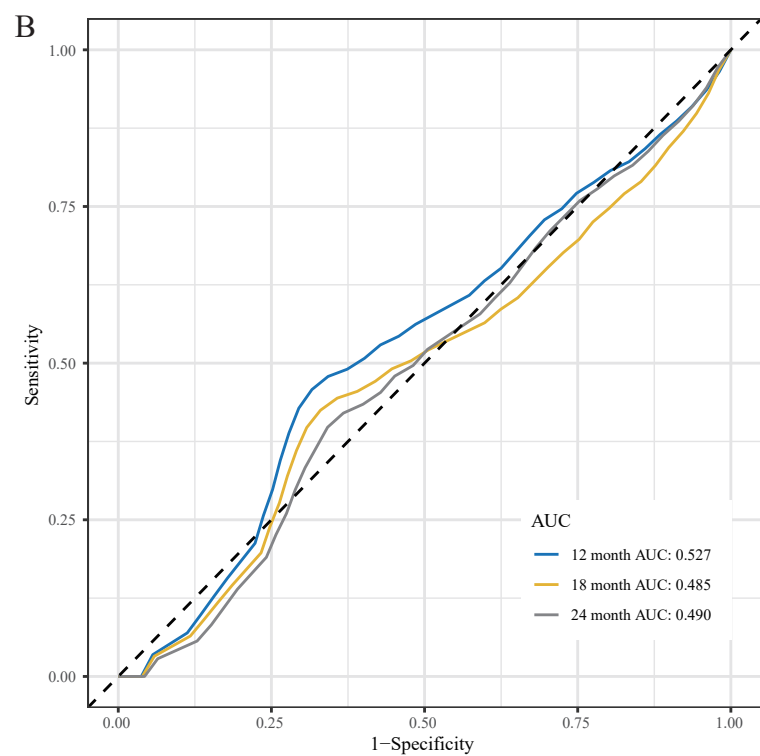

Supplement: Supplementary file 1 [file cells-10-00977-s001.zip › Zhou et al_R_Suppl Figure2.pdf]
